# Impact of Vitamin D Deficiency on COVID-19—A Prospective Analysis from the CovILD Registry

**DOI:** 10.3390/nu12092775

**Published:** 2020-09-11

**Authors:** Alex Pizzini, Magdalena Aichner, Sabina Sahanic, Anna Böhm, Alexander Egger, Gregor Hoermann, Katharina Kurz, Gerlig Widmann, Rosa Bellmann-Weiler, Günter Weiss, Ivan Tancevski, Thomas Sonnweber, Judith Löffler-Ragg

**Affiliations:** 1 Department of Internal Medicine II, Infectious Diseases, Pneumology, Rheumatology, Medical University of Innsbruck, 6020 Innsbruck, Austria; alex.pizzini@i-med.ac.at (A.P.); magdalena.aichner@student.i-med.ac.at (M.A.); sabina.sahanic@i-med.ac.at (S.S.); anna-k.boehm@student.i-med.ac.at (A.B.); katharina.kurz@i-med.ac.at (K.K.); rosa.bellmann-weiler@i-med.ac.at (R.B.-W.); guenter.weiss@i-med.ac.at (G.W.); ivan.tancevski@i-med.ac.at (I.T.); Judith.loeffler@i-med.ac.at (J.L.-R.); 2 Central Institute of Medical and Chemical Laboratory Diagnostics, Medical University of Innsbruck, 6020 Innsbruck, Austria; alexander.egger@tirol-kliniken.at (A.E.); gregor.hoermann@tirol-kliniken.at (G.H.); 3 Department of Laboratory Medicine, Medical University of Vienna, 1090 Vienna, Austria; 4 MLL Munich Leukemia Laboratory, Klinikum Großhadern, 81377 Munich, Germany; 5 Department of Radiology, Medical University of Innsbruck, 6020 Innsbruck, Austria; gerlig.widmann@tirol-kliniken.at; 6 Christian Doppler Laboratory for Iron Metabolism and Anemia Research, Medical University of Innsbruck, 6020 Innsbruck, Austria

**Keywords:** vitamin D, VITD, COVID-19, SARS-CoV-2, PTH, parathyroid hormone

## Abstract

The novel Coronavirus disease 2019 (COVID-19) caused by severe acute respiratory syndrome coronavirus type 2 (SARS-CoV-2) is a global health concern. Vitamin D (VITD) deficiency has been suggested to alter SARS-CoV-2 susceptibility and the course of disease. Thus, we aimed to investigate associations of VITD status to disease presentation within the CovILD registry. This prospective, multicenter, observational study on long-term sequelae includes patients with COVID-19 after hospitalization or outpatients with persistent symptoms. Eight weeks after PCR confirmed diagnosis, a detailed questionnaire, a clinical examination, and laboratory testing, including VITD status, were evaluated. Furthermore, available laboratory specimens close to hospital admission were used to retrospectively analyze 25-hydroxyvitamin D levels at disease onset. A total of 109 patients were included in the analysis (60% males, 40% females), aged 58 ± 14 years. Eight weeks after the onset of COVID-19, a high proportion of patients presented with impaired VITD metabolism and elevated parathyroid hormone (PTH) levels. PTH concentrations were increased in patients who needed intensive care unit (ICU) treatment, while VITD levels were not significantly different between disease severity groups. Low VITD levels at disease onset or at eight-week follow-up were not related to persistent symptom burden, lung function impairment, ongoing inflammation, or more severe CT abnormalities. VITD deficiency is frequent among COVID-19 patients but not associated with disease outcomes. However, individuals with severe disease display a disturbed parathyroid-vitamin-D axis within their recovery phase. The proposed significance of VITD supplementation in the clinical management of COVID-19 remains elusive.

## 1. Introduction

The novel Coronavirus disease 2019 (COVID-19) caused by severe acute respiratory syndrome coronavirus type 2 (SARS-CoV-2) is a global health concern leading to a substantial need for patient hospitalization, treatment at intensive care units (ICUs), and invasive ventilation [1,2,3]. There is a risk of morbidity and mortality from COVID-19 as a consequence of severe pulmonary involvement and multi-organ failure varies across the general population. Several risk factors impacting the clinical course of COVID-19 have been described in the literature [4,5,6,7,8], and in most of these conditions, vitamin D (VITD) deficiency occurs frequently, especially in advanced age [9]. 

VITD, traditionally known as a crucial regulator of bone metabolism, is obtained either from nutritional sources or endogenous production. The endogenous generation of 25-hydroxyvitamin D (25(OH)D), the major circulating form of VITD, includes various enzymatic steps. One crucial step in 25(OH)D production, the transformation of 7-dehydrocholesterol to previtamin D3, takes place in the skin and depends on the action of UV light intensity and duration [10]. Thus, VITD deficiency is related to seasonal changes, being most prevalent during winter season, and is very common in general, especially in developed countries. Ultimately, the fully active hormone is generated by hydroxylation at position 1 in the kidney to form 1,25-dihydroxyvitamin D (1,25(OH)D) [11]. This final step is catalyzed by CYP27B1, an enzyme also found in many extrarenal tissues, which are able to produce 1,25(OH)D in a para- or autocrine manner [12].

As suggested by the Task Force of the Endocrine Society, VITD deficiency is determined by measurement of serum 25(OH)D, which represents the most robust indicator to monitor the VITD status [13]. Accordingly, VITD deficiency is defined by a serum 25(OH)D concentration below 30 nmol/L, whereas serum 25(OH)D concentration below 50 nmol/L indicates insufficient VITD supply. Based on these definitions, up to 40 percent of European individuals suffer from VITD deficiency [14,15].

Besides, various non-skeletal functions have been associated with VITD metabolism [16]. For instance, VITD interacts with the RAAS system, thus altering vascular wall tension and blood pressure, and may prevent the establishment and progression of atherosclerosis [17,18]. Additionally, VITD is related to immune surveillance and contributes to defense against bacterial and viral infections [19,20]. In this context, previous studies revealed a higher susceptibility to seasonal influenza and respiratory syncytial virus infections in VITD-deficient subjects [20,21]. VITD deficiency was further related to cases of severe pneumonia and the development of acute lung injury [22]. Since these conditions may occur as clinical features of a SARS-CoV-2 infection, we scrutinized the impact of VITD metabolism on the clinical course of COVID-19. The potential benefits of normal-ranged VITD levels in COVID-19 have previously been suggested; however, published studies on the impact of VITD status on the course of COVID-19 are lacking [20]. Moreover, public interest in VITD seems to be even more significant since the SARS-CoV-2 outbreak. Google trends revealed a significant boost in the frequency of “Vitamin D” being looked up during the SARS-CoV-2 pandemic [23]. Thus, we aimed to analyze VITD status and its associations with clinical presentation and course of disease in COVID-19.

## 2. Materials and Methods

Herein, we report results of the ongoing prospective multicenter observational CovILD study (ClinicalTrials.gov number, NCT04416100), aiming to evaluate the persistent cardio-pulmonary damage of COVID-19 patients. This prospective, multicentre, observational study includes patients with a confirmed diagnosis of COVID-19, based on typical clinical presentation and a positive SARS-CoV-2 real-time PCR test. The target population included hospitalized patients as well as outpatients with persistent symptoms. Inclusion criteria were female and male patients ≥18 years with a confirmed infection with SARS-CoV-2 according to the definition of the Austrian Federal Ministry of Social Affairs, Health, Care, and Consumer Protection, and signed and dated declaration of consent by the patient according to ICH-GCP Guidelines [24]. Exclusion criteria were pregnancy, dementia, or declaration of consent by the patient according to ICH-GCP Guidelines not signed. 

Enrollment of patients with confirmed SARS-CoV-2 infection began on 29 April 2020. The trial site was located in Innsbruck, with two additional participating centers in Zams and Münster, all care centers in Tyrol, the first major COVID-19 hotspot in Austria. Due to the sudden rise in infections, patients had to be treated in wards from different medical disciplines. In total, the study cohort included 22 outward and 87 hospitalized patients, of whom 18 patients needed treatment at the ICU. Eight weeks after the confirmed diagnosis, a detailed questionnaire, clinical examination; and lung function testing including spirometry, body plethysmography, exhaled nitric oxide (FeNO), diffusion capacity for carbon monoxide adjusted for haemoglobin (DLCOc), capillary blood gas analysis, trans-thoracic echocardiography, standard laboratory examinations, a low-dose computed tomography (CT) scan of the chest, and laboratory testing, were evaluated. Laboratory parameters relevant for this analysis included 25(OH)D (nmol/L), parathyroid hormone (PTH) (ng/L), calcium (total and ionized, nmol/L), phosphate (mmol/L), creatinine (mg/dL), urea (mg/dL), C-reactive-protein (CRP), interleukin-6 (IL-6), serum ferritin, and D-dimer. Additionally, specimens of 37 patients acquired during the first days of hospital admission were used to retrospectively determine 25(OH) levels at disease onset. According to current guidelines, VITD deficiency was defined as 25(OH)D levels below 30 nmol/L. Serum concentrations of 25(OH)D between 30 and 50 nmol/L were categorized as insufficient VITD supply, whereas 25(OH)D above 100 nmol/L were considered normal [12]. Disease severity was categorized as mild for patients in outward treatment; moderate for patients in inward treatment; and severe for patients requiring oxygen supply, respiratory support, or intensive care treatment.

CT images were evaluated for the presence of ground-glass opacities (GGO), consolidations, bronchiectasis, and reticulations, as defined by the glossary of terms of the Fleischner society, and the intensity of the findings was graded according to their distribution (unilateral/bilateral, involved lobes) by assigning a score ranging from 0–5 per lobe [25].

Mean comparison of normally distributed numeric data was performed using Student’s *t*-test. If Gaussian distribution was not given, the Mann–Whitney-U-test and Kruskal–Wallis-test were applied. Spearman rank correlation coefficient was used for the analysis of monotonic associations in non-normally distributed data. If Gaussian distribution was given, Pearson correlation coefficient was calculated to assess the degree of linear associations. All tests were calculated two-tailed, and a *p*-value of 0.05 indicated statistical significance. Statistical analyses were performed with SPSS 24.0 statistical package (IBM Corp., Armonk, NY, USA). 

All procedures performed in the present study involving human participants were in accordance with the ethical standards of the Institutional and/or National Research Committee and with the 1964 Helsinki declaration and its later amendments, and were performed after approval of the ethics committee of the Medical University of Innsbruck (EK Nr: 1103/2020).

## 3. Results

The study cohort consisted of predominantly male individuals (60%), aged 58 ± 14 years. Sixty-five percent of COVID-19 patients were overweight or obese, and most individuals had pre-existing comorbidities, with cardiovascular and endocrine diseases being the most frequent ones. 

During hospitalization, most patients needed oxygen supply (53%), and 21 percent were admitted to the ICU due to the necessity of non-invasive or invasive mechanical ventilation as determined by the treating physicians. Detailed demographics and clinical characteristics of the study cohort are shown in Table 1.

**Figure 1:**
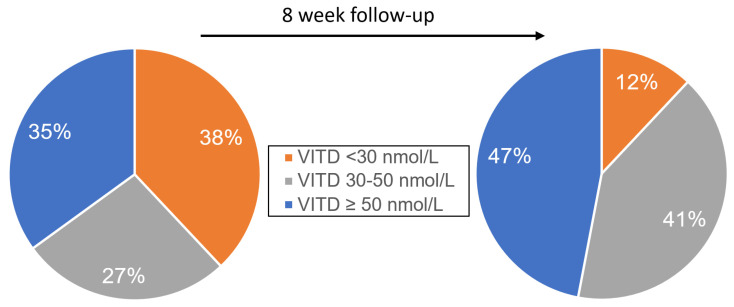
Vitamin D during hospitalization and after 8-week follow-up. Data is presented as percentage and categorized into Vitamin D (VITD) concentrations <30 nmol/L, 30–50 nmol/L, and >50 nmol/L. On the left, data during hospitalization is compared to data from 8-week follow-up on the right.

Eight weeks after onset of COVID-19, mean 25(OH)D concentrations were 54 ± 25 nmol/L, demonstrating a high proportion of patients with impaired VITD metabolism, and highlighting male patients, who displayed significantly lower 25(OH)D levels (50 nmol/L ± 23 vs. 61 ± 25 nmol/L, *p* = 0.01) than females. Overall, 12% of patients presented with VITD deficiency and 41% with VITD insufficiency. Accordingly, PTH was significantly increased (>65 ng/L) in 13 percent of patients. From 37 patients, 25(OH)D levels could be retrospectively assessed from samples stored at the moment of COIVD-19 diagnosis. The mean 25(OH)D concentration was 49 ± 36 nmol/L. During hospitalization, 38% of patients had 25(OH)D deficiency and 27% 25(OH) insufficiency (Figure 1).

The 25(OH)D concentrations at diagnosis highly correlated with those at the first follow-up visit (r = 0.805, *p* < 0.01), and significant rise in 25(OH)D concentrations was noted during the observation period (*p* = 0.01). 

**Figure 2:**
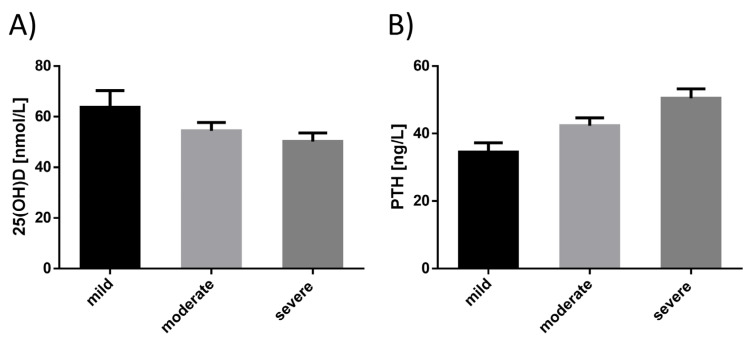
Vitamin D (**A**) and parathyroid hormone (PTH) (**B**) concentration according to disease severity at eight weeks follow-up: serum concentrations of (**A**) 25-hydroxyvitamin D (25(OH)D) and (**B**) PTH. Disease severity was graded according to intensity of treatment: mild = ambulatory treatment; moderate = hospital treatment; severe=inward treatment with respiratory (oxygen) supply or treatment at the ICU with non-invasive or invasive ventilation.

PTH concentrations were more significantly elevated in subjects who needed ICU or prolonged oxygen treatment (*p* < 0.01) than in mild cases. Similar differences were noted when comparing 25(OH)D levels among mild, moderate, and severe groups, although they did not reach statistical significance. Still, when comparing 25(OH)D levels of the severe subgroup to pooled data of mild and moderate cases, they were significantly lower (58 ± 25 vs. 50 ± 24 nmol/L, *p* < 0.05) at follow-up. In contrast, 25(OH)D concentrations at disease onset did not differ significantly between these groups. (*p* = 0.20). (*p* = 0.12; Figure 2, Table 2). 

When we compared 25(OH)D levels in patients with or without CT abnormalities, no significant difference was apparent (55nmol/L vs. 54 nmol/L, *p* = 0.83). Accordingly, 25(OH)D concentrations in patients with normal or impaired lung function did not show significant differences either at disease onset (48 vs. 50 nmol/L, *p* = 0.84) or at follow-up (57 vs. 50 nmol/L, *p* = 0.15), and 25(OH)D levels at disease onset did not predict symptom burden at follow-up. The same was the case when analyzing PTH levels in regards to CT abnormalities (*p* = 0.07), impaired lung function (*p* = 0.89), and persistent symptoms (*p* = 0.82). Correlation analysis between 25(OH)D levels at follow-up and CRP (r = −0.021, *p* = 0.88), IL-6 (r = −0.003, *p* = 0.98), serum ferritin (r = 0.019, *p* = 0.89), and D-dimer (r = −0.155, *p* = 0.26) revealed no significant associations. The same was true for 25(OH)D levels at disease onset and CRP (r = 0.152, *p* = 0.45), IL-6 (r = 0.050, *p* = 0.80), and serum ferritin (r = 0.070, *p* = 0.73). In contrast, D-Dimer levels were moderately associated with 25(OH)D levels at disease onset (r = 0.437, *p* < 0.05). 

Ten (9.2 %) of patients received VITD supplementation during hospitalization, but the initiation of VITD supplementation was not related to disease severity.

## 4. Discussion

The herein-presented analysis of the CovILD study cohort shows that 25(OH)D deficiency is common among COVID-19 patients, whereas a causal implication of VitD metabolism on its disease course remains uncertain. 

Evidence from a study by Hastie et al., who retrospectively analyzed the influence of vitamin D status on COVID-19 infection risk, supports the here presented results. Comparison of 25(OH)D levels of COVID-19 patients with UK Biobank data did not support a potential role for VITD metabolism for the susceptibility to COVID-19 infection nor for the differences between ethnic groups [26]. As pointed out in the response by Roy et al., the study focused on the association of VITD to the risk of incidence of COVID-19 rather than the risk of severity [27]. Contrasting results are reported from an Israeli population-based study, which, similar to this study, identified a high frequency of 25(OH)D deficiency in COVID-19 patients. Multivariate analysis, after controlling for demographic characteristics and medical conditions, confirmed an independent and significant association between a low 25(OH)D level and an increased likelihood of COVID-19 infection [28]. 

This prospective observational cohort study reveals first evidence of a disturbed PTH–VITD axis in patients with a more severe course of COVID-19. Low levels of 25(OH)D, however, did not predict the severity of the disease and did not associate with persistent symptoms, CT-abnormalities, or impaired pulmonary function testing, either at the moment of COVID-19 diagnosis or at the 8-week follow-up. Nevertheless, the high proportion of patients with elevated PTH concentrations, especially in severe COVID-19 cases during the recovery phase, is remarkable. This might simply reflect the result of less sunlight exposure as a consequence of prolonged quarantine periods and hospitalization, leading to secondary hyperparathyroidism, or represent a residual dysregulation after the infectious disease [29]. Patients’ reduced mobility due to persistent COVID-19 related symptoms such as fatigue and dyspnoea may also contribute to the described alterations. The recently observed peaks in new SARS-CoV-2 infections in regions with high sunlight exposure and consequently expected abundant endogenous VITD synthesis, like Florida and California, further questions the significance of VITD on COVID-19 susceptibility and disease course, contrasting with the so-called latitude hypothesis [30]. 

Results from a small Italian study including 42 COVID-19 patients with respiratory failure treated at the ICU identified 81% of the patients having 25(OH)D deficiency. Severe deficiency, defined as 25(OH)D below 10 ng/mL, was identified in 10 patients and associated with a significant elevation in mortality, despite being by far the oldest study-subgroup with comorbidities in every patient. A direct comparison with our study is inappropriate as study-design and outcome-measurements distinctly differ; however, a proper validation in a larger cohort of ICU patients is warranted in order to draw robust conclusions related to the prognostic impact of VITD deficiency in critically ill COVID-19 patients.

The absolute increase in serum 25(OH)D levels from the time of COVID-19 diagnosis to follow-up is subject for discussion. As 25(OH)D levels were not routinely analyzed in COVID-19 patients during hospitalization, we used laboratory specimens close to hospital admission to retrospectively determine 25(OH)D levels. Still, laboratory specimens were only available from a subgroup of the cohort (*n* = 37), thus results of this analysis have to be interpreted with caution. Additionally, patients with a severe lack of 25(OH)D received VITD supplementation as part of the clinical management.

The immunomodulatory effects of VITD described in the context of pneumonia, acute lung injury, and systemic inflammatory response syndrome would sustain a potential link to COVID-19 and imply a possibility for intervention [31]. The known interaction of VITD with the renin-angiotensin system, including ACE2, whose receptor SARS-Cov-2 binds to, further recommends it as a potential therapeutic option [32]. Accordingly, in a rat model, VITD has already been shown to alleviate acute lung injury by modulating the renin-angiotensin system [33]. However, previous reports also question the causal implication of VITD deficiency, especially low 25(OH)D levels, in extra-skeletal health [29,34] by discussing the potential ability of infections and chronic inflammatory processes in reducing serum 25(OH)D levels. Such causal association would also explain why VITD insufficiency/deficiency is reported in a wide range of disorders, although results of randomized controlled trials using vitamin D supplementation in preventing or ameliorating extra-skeletal diseases are mostly inconsistent or even disappointing [34,35]. 

Although the abovementioned studies justify further analyses, including 25(OH)D interventional studies, in COVID-19, our results do not support VITD deficiency as a strong indicator of severe disease course. However, as the CovILD study was not designed to evaluate the effects of 25(OH)D in COVID-19, we have to acknowledge some limitations. First, the CovILD trial was designed as an observational study to detect the rate of persistent lung injury after a severe course of COVID-19, therefore the here presented results must be interpreted carefully in the context of VITD deficiency, especially because predominantly patients with severe disease course were included. Second, 25(OH)D levels were prospectively assessed eight weeks after the positive SARS-Cov-2 PCR test, while only a third of the study population 25(OH)D levels at disease onset were retrospectively available. Thus, further prospective clinical studies are needed to clarify the significance of VITD in the clinical management of COVID-19 patients. 

## 5. Conclusions

VITD deficiency is frequently found in patients with severe COVID-19, but 25(OH)D concentrations do not associate with persistent inflammation, impairment in pulmonary function tests, pathological findings in CT-scans, or the persistence of symptoms. However, individuals with severe disease display a disturbed parathyroid-vitamin-D axis within their recovery phase, most likely due to prolonged hospitalization, although the question about causality or consequence cannot be answered through our data, and more evidence from interventional RCTs is warranted to properly understand the role of VITD in COVID-19. Conclusively, the proposed significance of VITD in the clinical management of COVID-19 remains elusive. 

## Figures and Tables

**Table 1:** Demographics and clinical characteristics of patients with COVID-19.

Characteristics	(*N* = 109)
Median age (SD)–yr	58 ± 14
Female sex–no. (%)	44 (40)
Mean body mass index–kg/m^2^ (SD) *	27 ± 14
**Comorbidities No. (%)**	
None	21 (19)
Cardiovascular disease	44 (40)
Hypertension	32 (29)
Pulmonary disease	21 (19)
Endocrine disease	49 (45)
Hypercholesterolemia	24 (22)
Diabetes, type 2	20 (18)
Chronic kidney disease	7 (6)
Chronic liver disease	6 (6)
Malignancy	16 (15)
Immunodeficiency	7 (6)
**Treatment**	
Oxygen supply–no. (%)	53 (49)
Non-invasive ventilation–no. (%)	2 (2)
Invasive ventilation–no. (%)	16 (15)
Vitamin-D supplementation	10 (9)

* The body-mass index (BMI) is the weight kilograms divided by the square of the height in meters.

**Table 2:** Laboratory parameters in COVID-19 patients 8 weeks after disease onset.

	Total (*N* = 109)	Mild (*N* = 22)	Moderate (*N* = 34)	Severe * (*N* = 53)	*p*-Value
Median age (SD)–yr	58 ± 14	46 ± 16	60 ± 13	61 ± 12	0.001
Female sex–no. (%)	44 (40)	14 (64)	20 (60)	10 (19)	0.001
Mean BMI–kg/m^2^ (SD) †	27 ± 14	26 ± 5	26 ± 4	28 ± 5	0.287
Days of hospitalization	9 ± 10	0 (0)	5 ± 3	15 ± 10	<0.001
25(OH)D nmol∙L^−1^	54 ± 25	64 ± 31	54 ± 19	50 ± 24	0.116
PTH–ng∙L^−1^	45 ± 18	35 ± 13	42 ± 14	50 ± 20	0.001
Calcium–mmol∙L^−1^					
total	2.37 ± 0.09	2.37 ± 0.09	2.36 ± 0.09	2.39 ± 0.08	0.183
ionized	1.22 ± 0.04	1.24 ± 0.03	1.22 ± 0.04	1.22 ± 0.04	0.310
Phosphate–mmol∙L^−1^	1.01 ± 0.17	1.02 ± 0.14	1.04 ± 0.16	0.99 ± 0.19	0.473
Creatinine–mg∙dL^−1^	0.87 ± 0.23	0.82 ± 0.15	0.80 ± 0.17	0.93 ± 0.27	0.017
Urea–mg∙dL^−1^	32 ± 11	28 ± 7	32 ± 9	33 ± 13	0.242
CRP–mg∙dL^−1^	0.29 ± 0.44	0.2 ± 0.28	0.2 ± 0.21	0.39 ± 0.56	0.067
IL-6–ng∙L^−1^	3.1 ±4.98	1.45 ± 2.06	1.96 ± 1.95	4.43 ± 6.6	0.041
D-dimer–ug∙L^−1^	807 ± 1591	607 ± 797	632 ± 633	1001 ± 2160	0.475
Ferritin–ug∙L^−1^	263 ± 230	139 ± 118	260 ± 183	317 ± 271	0.001

† BMI, body-mass index; the BMI is the weight kilograms divided by the square of the height in meters. * 35 patients received oxygen supply only, two patients were treated with non-invasive ventilation, and 16 with invasive ventilation. Disease severity was graded according to intensity of treatment: mild = ambulatory treatment; moderate = hospital treatment; severe = inward treatment with respiratory (oxygen) supply or treatment at the intensive care unit (ICU) with non-invasive or invasive ventilation; PTH = parathyroid hormone; CRP = C-reactive protein; IL-6 = interleukin 6; data are depicted as mean ± SD, p-values were calculated with Kruskal–Wallis Test.
